# Impact of Time-Varying Treatment Exposures on the Risk of Venous Thromboembolism in Multiple Myeloma

**DOI:** 10.3390/healthcare4040093

**Published:** 2016-12-20

**Authors:** Joshua D. Brown, Val R. Adams, Daniela C. Moga

**Affiliations:** 1 Department of Pharmacy Practice and Science, University of Kentucky College of Pharmacy, Lexington, KY 40536, USA; val.adams@uky.edu (V.R.A.); daniela.moga@uky.edu (D.C.M.); 2 Department of Pharmaceutical Outcomes & Policy, University of Florida College of Pharmacy, Gainesville, FL 32610, USA

**Keywords:** multiple myeloma, venous thromboembolism, deep vein thrombosis, competing risks, case-time-control study design

## Abstract

Multiple myeloma (MM) has one of the highest risks of venous thromboembolism (VTE) of all cancers due to pathologic changes and treatment-related exposures. This study assessed the one-year incidence of VTE in newly diagnosed MM and to determine the baseline and time-varying treatment-related factors associated with VTE risk in a U.S.-based cohort. MM patients were identified and age, gender, and baseline comorbidities were determined. Treatment-related exposures included thalidomide derivatives (IMIDs), proteasome inhibitors, cytotoxic chemotherapy, steroids, erythropoietin-stimulating agents (ESAs), stem cell transplants (SCT), hospitalizations, infection, and central venous catheters (CVC). Multiple statistical models were used including a baseline competing risks model, a time-varying exposure Cox proportional hazard (CPH) model, and a case-time-control analysis. The overall incidence of VTE was 107.2 per 1000 person-years with one-half of the VTEs occurring in the first 90 days. The baseline model showed that increasing age, heart failure, and hypertension were associated with one-year incidence of VTE. MM-specific IMID treatment had lower than expected associations with VTE based on prior literature. Instead, exposure to ESAs, SCT, CVC, and infection had higher associations. Based on these results, VTE risk in MM may be less straightforward than considering only chemotherapy exposures, and other treatment-related exposures should be considered to determine patient risk.

## 1. Introduction

Multiple myeloma (MM) has one of the highest risks of thrombosis among all cancers due to disease-related pathological changes and treatment [1,2,3]. Malignancy induces a prothrombotic state, which includes activation of the coagulation cascade, increase in pro-inflammatory cytokines, and inhibition of natural anticoagulants [3,4,5]. This is further exacerbated by cancer treatment and surgery. Thalidomide and lenalidomide (IMIDs) are well known to be associated with increased risk of thrombosis [6], especially when combined with high-dose steroids and other chemotherapy, with incidence approaching 25% in some studies [7,8,9,10]. Other common MM treatments include proteasome inhibitors (PIs; bortezomib, carfilzomib) and cytotoxic therapies (cyclophosphamide, melphalan, others), which have been shown to have a lower, though still increased, risk of venous thromboembolism (VTE) compared to IMIDs [11]. Other disease-related factors with potential to increase thrombotic risk include use of central venous catheters (CVC), erythropoietin-stimulating agents (ESAs), hospitalization, and infection [5,12,13,14,15,16,17,18]. Due to this inherent increased risk of thrombosis with MM, guidelines recommend routine thromboprophylaxis with anticoagulants (low-molecular weight heparins (LMWH), warfarin, etc.) or aspirin especially among those receiving IMIDs with steroids [19,20].

Previous studies have assessed VTE risk in MM during randomized-controlled trials (RCTs) or in small observational studies with limited information about other risk factors associated with MM-related thrombosis [8,9,10,21]. These studies have also considered static treatment, not taking into account the time-varying nature of chemotherapy regimens and other disease-related exposures that may have an acute impact on thrombosis risk, e.g., supportive therapies [12,14,15,16,17,18]. As these exposures are potentially modifiable or detectable, identifying high-risk exposures may lead to better prediction of thrombotic events and lead to enhanced surveillance or prevention efforts.

The objective of this study was to determine the one-year incidence of VTE in newly diagnosed MM, assess the association of baseline characteristics and thrombosis, and to investigate the acute association between disease-related treatments and exposures with thrombosis. While previous studies have shown increased risks associated with specific treatments, our a priori hypothesis was that other treatment exposures occurring during treatment, such as supportive therapy or hospitalizations, may be attributing the observed increased risk of thrombosis associated with MM.

## 2. Methods

### 2.1. Data Source and Study Sample

This was a retrospective cohort study utilizing an extract of patients with at least two diagnoses of MM (International Classification of Diseases codes, 9th Revision (ICD-9): 203.0x) at least 14 days apart during 2008–2013 from the Truven MarketScan Commercial Claims and Encounters and Medicare Supplemental databases. The MarketScan data are administrative claims data including medical diagnostic and procedural billing information and pharmacy fill records for those with commercial insurance linked to demographic and insurance enrollment information for each individual. The dataset represents patients from all 50 U.S. states and is representative based on demographic and geographic characteristics. The data do not include detailed clinical information (e.g., laboratory values or cancer stating) but are a complete record of a patient’s healthcare utilization.

For further inclusion, subjects were required to have a minimum of six months of continuous medical and pharmacy insurance coverage prior to the first MM diagnosis and be at least 18 years or older at diagnosis. Subjects also could not have a previous diagnosis of another cancer or a thrombotic outcome event during the six-month, pre-index period. Use of the MarketScan data was approved by the University of Kentucky Institutional Review Board (15-0334-P6A).

### 2.2. Subject Characteristics

Age was assessed on the MM index date and gender was linked from the enrollment file. The Charlson Comorbidity Index was used to assess comorbidity burden based on the ICD-9 coding algorithm by Quan et al. [22] and the total score was further categorized by 0, 1–2, 3–4, and 5+ groups with individual comorbidities also reported. Anemia in the pre-index period was also assessed using ICD-9 codes.

### 2.3. Outcome Events

Deep vein thrombosis (DVT) and pulmonary embolism (PE) VTE events were assessed based on previously published ICD-9 algorithms [23,24,25]. Date of death was based on discharge status codes on hospital or hospice records. Loss to follow-up occurred when continuous insurance coverage ended during the follow-up period or follow-up terminated at the end of the data (December 2013). All other individuals were censored after one year of follow-up. If a thrombosis occurred on the same day as death, the event was recorded as the thrombosis as it was the main outcome of interest in this study. Subjects were followed in this retrospective cohort until one of the following occurred: (1) a thrombosis event; (2) death; (3) loss to follow-up; or (4) end of the one-year study period.

### 2.4. Statistical Analysis

Person time was calculated correcting for the differential follow-up of each subject. The incidence rate of VTE was reported as the rate per 1000 person-years. Incidence rates were calculated at several intervals (30, 60, 90, 180 days) as well as for the complete one-year study period.

Due to the high risk of death associated with MM as well as the baseline risk of death in the older population, it was necessary to assess thrombosis alongside the competing risk of death over longer follow-up periods [26]. In a survival analytic framework, death prevents the occurrence of an outcome of interest; thus, death cannot simply be considered a censored observation [27]. However, there is no statistical framework to assess competing risks with time-varying covariates. Utilization of a traditional Cox proportional hazard (CPH) model in this example, which allows for time-varying exposures, would have produced bias estimates of the association especially as the cumulative incidence of death increases over the one-year period [26]. Thus, three statistical modeling approaches were used to investigate the influence of baseline, time-independent factors as well as time-dependent, treatment-related exposures separately.

#### 2.4.1. Model #1—Competing Risks Survival Analysis

The association of VTE with baseline demographic and clinical characteristics at diagnosis was assessed using a competing risks regression model using the complete follow-up time for each individual up to one year of follow-up. Post-diagnosis, treatment-related covariates were excluded from this model to avoid the bias of ignoring competing risks. In this model, the dependent outcome has three levels: 0 = censored; 1 = thrombosis; and 2 = death. Subdistribution hazard ratios (HR) and their 95% confidence intervals (CI) for the association between each baseline covariate and thrombotic events were estimated for each baseline covariate included in the model. A post-hoc analysis restricted the model to the first 90 days after diagnosis to explore the association in the early post-diagnosis phase, which showed no significant differences from the main model. The one-year cumulative incidence of thrombotic events was reported for the total cohort.

#### 2.4.2. Model #2—Case-Time-Control Analysis

The case-time-control study design incorporates both the case-control and case-crossover study designs. Since it assigns cases by outcome events, it is not sensitive to the bias associated with competing risks. The case-time-control study design assumes that if an exposure causes a transient increase in the risk of an event then it will be more common in the “hazard” period immediately before an event; defined for this study as the 30 days immediately proximal to the event. A washout period was used between days 31–60 and the comparison period consisted of days 61–90 before the event [28]. Cases included 502 VTEs, with at least a 90-day pre-event exposure window to allow for the hazard, washout, and comparison periods. Each case was matched with up to four controls based on the exact year of MM diagnosis and who had overlapping enrollment periods using incidence density sampling with replacement. Matching gave 2008 matched pairs with 1732 unique controls. Exposures in the hazard and comparison periods are compared for each matched pair and adjusted odds ratios (OR) are reported. Control subjects serve to control for the exposure trends over time rather than individual characteristics [28]. All demographic and time-independent characteristics, as well as unobserved confounders, are self-controlled and not included in the regression [28,29].

Exposures were assessed as binary indicators in each exposure period and included: IMIDs (thalidomide, lenalidomide, pomalidomide); PIs (bortezomib, carfilzomib); steroids (dexamethasone, prednisone, prednisolone); cytotoxic chemotherapy (doxorubicin, cyclophosphamide, melphalan, vincristine, etoposide, cisplatin); stem cell transplant (SCT); hospitalization (other than SCT-related hospitalizations); colony stimulating factors (CSF); central venous catheter (CVC); ESAs; and infection. Infection was broadly coded to encompass all bacterial, viral, and fungal infections. Because anticoagulation was highly collinear with IMID treatment, it was not assessed in any of the models. Conditional logistic regression was used to estimate the adjusted case-time-control OR and 95% CIs were calculated by bootstrapping the sample with 5000 replications [28].

Sensitivity analyses were conducted varying the hazard, washout, and comparison exposure windows, which showed that the results were not sensitive to the specification. Further, for both Model #2 and Model #3 below, MM-specific treatments were categorized based on typical regimens (e.g., IMID + PI, IMID + steroid, PI + steroid, etc.) as well as separating the IMID group into individual products (thalidomide, lenalidomide, pomalidomide). The findings were not sensitive to these specifications.

#### 2.4.3. Model #3—Time-Varying Cox Proportional Hazard Models

Our previous work showed a high rate of thromboembolism associated with MM in the first 90 days after diagnosis [26]. Higher risk in the early stages of diagnosis and treatment may be related to high tumor burden and release of thrombogenic factors with initiation of treatment [7,30,31]. During this initial follow-up, mortality is lower; thus, the bias introduced by ignoring competing risks will be lower. Thus, a Cox proportional hazard (CPH) regression was also modeled to investigate the association between baseline factors as well as time-varying treatments and treatment-related exposures following patients for only the first 90 days after diagnosis.

All exposures from the case-time-control analysis were modeled as time-varying exposures by daily intervals. A residual risk period was programmed for each exposure to account for the prolonged increase in risk of thromboembolism event after an exposure occurs. Two models were estimated using 7 and 14 days for this risk window, which was added to the total exposure window. For outpatient prescription products, this included the dispensing date, days supplied, and the 7- or 14-day period. Inpatient chemotherapies and diagnoses included the day of exposure plus the additional risk window. Hospitalizations (SCT and non-SCT) included the day of admission, length of stay, and the risk window.

Sensitivity analyses for the CPH model included stratifying by exposure to prophylactic anticoagulation and whether or not MM treatment was initiated within this initial 90-day period. All baseline characteristics were included in the model and the proportionality assumption was tested for each. HRs and 95% confidence intervals were reported for all time-varying exposures. All analyses were conducted using SAS version 9.4 (SAS Institute, Cary, NC, USA).

## 3. Results

### 3.1. Incidence of Thrombosis

There were 1050 thrombosis events observed in 13,700 individuals during the one-year follow-up. This included 756 DVTs (72% of events) and 294 PEs (28%). Nearly one-half (N = 520, 49.5% of events) occurred within the first 90 days after MM diagnosis. The cohort contributed 9791.4 person-years of follow-up time for a one-year incidence rate of thrombosis of 107.2 (95% CI, 100.0–113.9) events per 1000 person-years. The highest incidence of thrombosis was in the first 30 days with 251 events and an incidence rate of 234.2 (95% CI, 206.5–264.5) per 1000 person-years. The rate of thrombotic events decreased over the 60-, 90-, and 180-day intervals: 196.6 (95% CI, 178.3–216.4), 171.7 (95% CI, 157.4–187.0), 140.1 (95% CI, 130.5–150.1), per 1000 person-years, respectively. There were 384 deaths experienced as a competing risk and a total of 479 deaths during the study period with an incidence rate of 48.9 (95% CI, 44.7–53.5) deaths per 1000 person-years.

**Figure 1:**
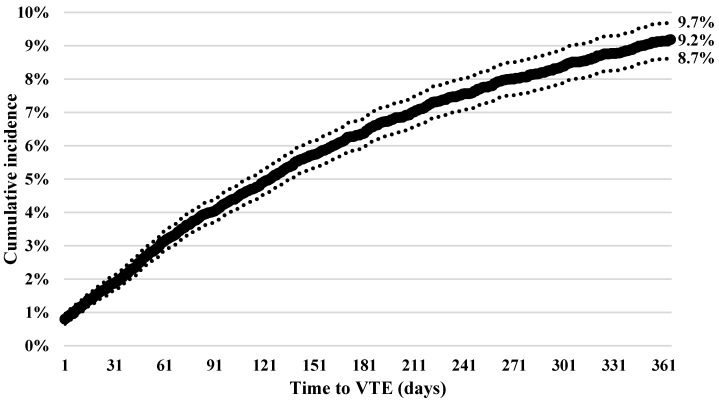
Cumulative incidence and 95% confidence interval of venous thromboembolism (VTE) from diagnosis of multiple myeloma.

The one-year cumulative incidence of thrombosis was 9.2% (95% CI, 8.7%–9.7%) for the total cohort (Figure 1). The cumulative incidence in the 18–34 age group was 5.6% (95% CI, 3.3%–9.6%) and 8.6% (95% CI, 7.9%–9.4%) in the 35–64 age group. There were no differences between the cumulative incidence of the 65–74 age group (10.2% (95% CI, 9.1%–11.5%)) and the 75 and older age group (9.9% (95% CI, 8.9%–11.1%)).

### 3.2. Competing Risk Model Results

Cohort baseline characteristics are summarized in Table 1. Among these characteristic in the baseline competing risk model, older age was associated with an increase in the hazard of thrombosis for the 35–64 and 65–74 age groups compared to the 18–34 reference group (Table 2). Female gender showed a protective effect with HR = 0.7 (95% CI, 0.7–0.8) compared to males. Increasing comorbidity burden had no impact on the hazard of thrombosis at baseline; however, some individual comorbidities at baseline did increase the risk. Those with congestive heart failure (CHF) had 70% higher hazard (HR = 1.7 (95% CI, 1.4–2.1)) and those with hypertension had 20% higher hazard (HR = 1.2 (95% CI, 1.0–1.3)). There were no other significant associations observed for the other included covariates. In the 90-day post-hoc analysis, the associations observed were nearly identical and included no additional significant associations compared to the primary, one-year model (results not shown).

### 3.3. Case-Time-Control Model

Table 3 shows the exposure profiles of cases and controls as well as the adjusted case-time-control OR for the odds of thrombosis with each exposure. IMID use was higher in the hazard period and had an adjusted OR of 1.61 (95% CI, 1.11–2.33), showing increased transient risk of IMIDs and thrombotic events. PI use was associated with lower odds (aOR = 0.65, (95% CI, 0.35–1.22)). The highest transient risk of thrombosis was shown for SCT (OR = 3.76, (95% CI, 3.07–4.61)), CVC (OR = 2.56, (95% CI, 2.28–2.87)), ESAs (OR = 3.82, (95% CI, 2.55–5.70)), and infection (OR = 2.51, (95% CI, 1.95–3.24)). All-cause hospitalization also had increased odds of thrombosis (OR = 1.24, (95% CI, 1.15–1.35)).

### 3.4. 90-Day Time-Varying Exposure Model

After adjustment for treatment exposures, there were no significant baseline characteristics associated with thromboembolism in the first 90 days after diagnosis. Among MM-specific chemotherapy, thalidomide (HR = 1.4, (95% CI, 1.1–1.8)) and steroids (HR = 1.5, (95% CI, 1.2–2.0)) had an increased hazard of thromboembolism in the first 90 days after diagnosis (Table 4). Stronger contributions were observed for infections (HR = 2.3, (95% CI, 1.8–3.0)), CVC use (HR = 2.0, (95% CI, 1.6–2.5)), and all-cause hospitalizations (HR = 8.9, (95% CI 7.3–11.0)). The directionality and magnitude of these findings were not sensitive to models including seven-day exposure windows or when stratified by those receiving anticoagulation or MM treatment in the 90-day period.

## 4. Discussion

Treatment advances over the last decade for MM have led to an increase in median survival greater than five years [32,33]. However, thrombotic complications have emerged as serious adverse effects of treatment; prompting recommendations for thromboprophylaxis in guidelines in this patient population [34,35]. Despite the known risk, the pathogenesis of thrombosis in MM is poorly understood due to the various factors that can impart risk including patient characteristics, disease-related factors, as well as treatment-related risks [3]. Although thrombotic events have been shown to have a negligible impact on overall survival in MM [33], thrombosis events can cause interruption in therapy as well as tremendous economic and humanistic burdens in the MM population [36,37]. Recent American Society of Clinical Oncology guidelines have called for better evidence regarding the increased risk of thrombosis and MM so that prevention efforts can be focused on periods of highest risk [38].

This study investigated both baseline and time-varying factors related to thrombosis risk in newly diagnosed MM. There were few baseline factors found to be associated with risk in our study, suggesting that risk may be associated with factors related to treatment of MM instead of pre-existing, patient-related factors. Further, these baseline characteristics were not significant after treatment was included in a time-varying CPH model. We observed that nearly one-half of all thrombotic events occurred within the first 90 days after MM diagnosis. This underscores the need to identify early risk factors at diagnosis to guide the utilization of thromboprophylaxis—especially in the first 90 days of treatment.

Treatment-related risks have been investigated in RCTs and small prospective studies using static treatment group assignment, and have not necessarily considered other disease-related exposures that may contribute to risk [3,10,11]. We considered several disease- and treatment-related factors including chemotherapy, supportive treatments, hospitalizations, and infections, which have all been shown to have increased risk of thrombosis in patients with cancer but have never been concurrently investigated [3]. Our findings suggest lower risks associated with chemotherapy regimens including IMIDs, PIs, steroids, and cytotoxic therapies than what may be expected based on prior literature [6,11]. Instead, the highest exposure associations were found for infections, CVC and hospitalizations. This suggests that simple association between treatment groups is not straightforward when considering risk and that other exposures, which may be downstream from the active chemotherapy treatment such as supportive therapies, may contribute more risk than previously acknowledged. This is strengthened by multiple methodologies showing similar results in this study.

Our study is subject to several limitations inherent to claims-based studies [39,40]. We relied on ICD-9 coding available in the claims to diagnose study subjects with outcome events and comorbidities. It is impossible to confirm a positive diagnosis using these data; however, claims-based coding algorithms for VTE have been shown to perform strongly especially when there is a high risk of VTE in the population [24,41]. Further, information regarding MM severity and staging is not available in claims data and, thus, could not be included here. This study is strengthened by use of statistical analyses to avoid potential shortfalls. We used a competing risks framework given that the outcome events cannot be considered independent of each other, i.e., experiencing one may preclude experiencing another or one event may cause another. Failure to do so can overestimate survival for traditional Kaplan-Meier or CPH analyses and lead to inflated cumulative incidence functions and biased associations [27]. In this study, this would have overestimated the cumulative incidence to be nearly 11% over the one-year study period and could bias any variables more strongly associated with death [26]. For this population, the competing risk of death is a contribution by many factors including the advanced age of the cohort, having cancer, as well as the risk of death from the other outcome events [27].

## 5. Conclusions

This study found an incidence rate of thrombotic events of 107.2 events per 1000 person-years in a U.S. cohort with multiple myeloma. Nearly one-half of all events occurred in the 90 days after multiple myeloma diagnosis. Other than IMIDs only, additional MM treatment-related risk factors for VTE included stem cell transplants, central venous catheters, ESAs, infection, and all-cause hospitalization. Consideration of high-risk exposures may guide clinicians to periods where increased surveillance or prophylactic interventions may be most impactful.

## Figures and Tables

**Table 1:** Baseline characteristics of the cohort, cases, and controls at diagnosis of multiple myeloma.

Overall Cohort N = 13,700		
	N	%
Age	Mean 63.9	SD 13.7
18–34	283	2.1
35–64	7389	53.9
65–74	2648	19.3
75+	3380	24.7
Gender		
Male	6625	48.4
Female	7075	51.6
Charlson Comorbidity Index	Mean 1.1	SD 1.5
0	6892	50.3
1–2	4758	34.7
3–4	1476	10.8
5+	574	4.2
Comorbidity		
MI	251	1.8
CHF	933	6.8
PVD	841	6.1
Dementia	123	0.9
COPD	1817	13.3
Rheumatism	734	5.4
PUD	134	1.0
Mild liver disease	551	4.0
Diabetes	2814	20.5
Diabetes w/complications	755	5.5
Paralysis	68	0.5
Renal disease	1826	13.3
Severe liver disease	45	0.3
CVD	910	6.6
HIV/AIDS	30	0.2
Hypertension	6466	47.2
CHD	1725	12.6
Lipids	4260	31.1
Anemia	3727	27.2

MI = myocardial infarction; CHF = congestive heart failure; PVD = peripheral vascular diseases; COPD = chronic obstructive pulmonary disease; PUD = peptic ulcer disease; CVD = cerebrovascular disease; CHD = coronary heart disease.

**Table 2:** Results of baseline competing risk survival analysis predicting venous thromboembolism outcome controlling for baseline factors.

Covariate	sHR	95% Confidence Interval	
Age			
18–34	Ref.	Ref.	Ref.
35–64 *	1.7	1.0	3.0
65–74 *	1.9	1.1	3.2
75+	1.6	0.9	2.7
Gender			
Male	Ref.	Ref.	Ref.
Female *	0.7	0.7	0.8
Charlson Comorbidity Index			
0	Ref.	Ref.	Ref.
1–2	1.0	0.5	2.1
3–4	0.8	0.5	1.3
5+	1.0	0.8	1.2
Comorbidities			
MI	0.9	0.6	1.4
CHF *	1.7	1.4	2.1
PVD	1.2	0.9	1.5
Dementia	1.2	0.7	2.1
COPD	0.9	0.8	1.1
Rheumatism	0.9	0.6	1.2
PUD	0.8	0.5	1.5
Mild liver disease	0.8	0.5	1.1
Diabetes	1.0	0.8	1.2
Diabetes with complications	1.1	0.8	1.5
Paralysis	1.4	0.8	2.5
Renal disease	1.0	0.8	1.3
Severe liver disease	1.1	0.4	3.0
CVD	1.0	0.8	1.3
Hypertension *	1.2	1.0	1.3
CHD	1.0	0.8	1.1
Lipids	1.0	0.9	1.1
Anemia	0.9	0.8	1.1

sHR = subdistribution hazard ratio; CI = confidence interval; MI = myocardial infarction; CHF = congestive heart failure; PUD = peptic ulcer disease; CVD = cerebrovascular disease; CHD = coronary heart disease; Ref. = Reference category; * *p* < 0.05.

**Table 3:** Results of case-time-control analysis predicting the time-varying association of treatment exposures and thrombosis.

Treatment/Exposure	Cases Exposure		Controls Exposure		CTC Adjusted Odds Ratio		
	Hazard N (%)	Comparison N (%)	Hazard N (%)	Comparison N (%)	Point Estimate	95% CI	
Thalidomide and derivatives	119 (23.7)	107 (21.3)	147 (8.5)	153 (8.8)	1.61	1.11	2.33
Proteasome inhibitors	108 (21.5)	121 (24.1)	140 (8.1)	180 (10.4)	0.65	0.35	1.22
Steroids	126 (25.1)	135 (26.9)	178 (10.3)	198 (11.4)	1.58	0.95	2.65
Cytotoxic chemotherapy	46 (9.2)	56 (11.2)	99 (5.7)	82 (4.7)	0.44	0.31	0.63
Stem cell transplant	28 (5.6)	11 (2.2)	15 (0.9)	18 (1.0)	3.76	3.07	4.61
Hospitalization	65 (13.0)	58 (11.6)	70 (4.0)	75 (4.3)	1.24	1.15	1.35
Colony stimulating factors	20 (4.0)	22 (4.4)	44 (2.5)	34 (2.0)	0.48	0.33	0.70
Central venous catheters	57 (11.4)	23 (4.6)	42 (2.4)	38 (2.2)	2.56	2.28	2.87
Erythropoietin-stimulating agents	44 (8.8)	27 (5.4)	47 (2.7)	47 (2.7)	3.82	2.55	5.70
Infection	74 (14.7)	45 (9.0)	90 (5.2)	87 (5.0)	2.51	1.95	3.24

CTC = case-time-control; CI = confidence interval.

**Table 4:** Cox proportional hazard model for VTE in the first 90 days after multiple myeloma diagnosis with time-varying exposures.

Time-Varying Exposures	HR	95% CI		HR	95% CI		HR	95% CI		HR	95% CI		HR	95% CI	
	Overall			Treated			Treated + Anticoagulation			Treated – Anticoagulation			Untreated		
Thalidomide derivatives	1.38	1.06	1.79	1.29	0.97	1.72	1.07	0.62	1.85	1.58	1.13	2.22	--	--	--
Proteasome Inhibitors	0.80	0.51	1.26	0.73	0.46	1.16	1.93	0.74	5.06	0.52	0.31	0.89	--	--	--
Steroids	1.54	1.21	1.96	2.18	1.60	2.95	2.81	1.43	5.55	2.05	1.45	2.90	--	--	--
Cytotoxic chemotherapy	1.15	0.76	1.73	1.37	0.89	2.10	0.80	0.31	2.07	1.61	0.99	2.61	--	--	--
Infections	2.29	1.80	2.92	1.88	1.28	2.77	1.42	0.49	4.13	1.84	1.21	2.79	2.15	1.55	2.98
Erythropoiesis-stimulating agents	1.03	0.64	1.67	1.08	0.63	1.87	1.09	0.37	3.20	1.33	0.71	2.49	*	*	*
Colony stimulating factors	0.93	0.43	1.99	1.09	0.50	2.39	2.28	0.47	10.94	0.93	0.37	2.33	*	*	*
Stem cell transplant	2.40	0.99	5.83	2.74	1.11	6.72	*	*	*	3.74	1.51	9.27	--	--	--
Central venous catheters	2.02	1.65	2.49	1.51	1.14	2.01	1.42	0.79	2.57	1.60	1.16	2.22	3.00	2.24	4.11
Hospitalization	8.90	7.26	10.92	7.16	5.40	9.50	1.50	0.78	2.90	12.02	8.57	16.84	15.72	11.52	21.44

HR = hazard ratio; CI = confidence interval; * Stable estimated could not be obtained due to low sample size. *Note:* All other covariates form Table 2 were included but were not significant predictors.
